# Evaluation of Colistin Susceptibility in Carbapenem‐Resistant *Acinetobacter baumannii* Isolates Using Broth Microdilution, MICRONAUT‐MIC‐Strip, and VITEK MS

**DOI:** 10.1002/mbo3.70046

**Published:** 2025-08-08

**Authors:** Fatih Mehmet Akıllı, Arzu İlki

**Affiliations:** ^1^ Faculty of Medicine, Department of Medical Microbiology Marmara University Istanbul Türkiye; ^2^ Sincan Training and Research Hospital Ankara Türkiye

**Keywords:** *Acinetobacter baumannii*, broth microdilution, carbapenems resistance, MALDI‐TOF MS, MALDIxin, MICRONAUT‐MIC‐Strip colistin assay

## Abstract

We aimed to analyze the colistin susceptibility using broth microdilution (BMD) and commercially available MICRONAUT‐MIC‐Strip (MMS) test and VITEK MS in carbapenem‐resistant *Acinetobacter baumannii* (CRAB) isolates. Colistin susceptibility of 194 CRAB isolated from various clinical specimens between December 2020 and 2022 was determined by BMD and commercial MMS method. Minimum inhibitory concentrations of the commercial method were compared with the standard BMD. In the second part of the study, colistin susceptibility of the isolates was tested using the MALDIxin method, which detects modified lipid A, by VITEK MS, MALDI‐TOF MS (bioMérieux, France). The presence of *mcr*‐1–5 genes in resistant isolates was investigated using in‐house multiplex polymerase chain reaction. Of these, 23 (11.8%) were found to be colistin resistant, whereas 171 (88.2%) isolates were susceptible. MMS categorical agreement was found to be 98.9% and essential agreement was 96.3%. The major error was found to be 1.03%, whereas a very major error was not detected. The MALDIxin test developed according to VITEK MS did not detect mutations responsible for lipopolysaccharide‐induced colistin resistance in our isolates (expected peak at 1935–2033 *m*/*z*). The mcr‐1–5 genes were not detected in our isolates. The MMS test is a reliable alternative method for the detection of colistin susceptibility in CRAB. Colistin resistance of the isolates used in our study was not associated with lipid A modification. Alternative mechanisms, such as efflux pumps, are a possibility. To our knowledge, this is the first VITEK MS–based method that enables rapid detection of colistin resistance in negative ion mode, and further studies are needed to determine colistin resistance in this way.

## Graphical abstract

The colistin susceptibility of 194 carbapenem‐resistant *Acinetobacter baumannii* isolates was assessed using broth microdilution (BMD), MICRONAUT‐MIC‐Strip (MMS), and VITEK MS–based MALDIxin methods. MMS demonstrated high categorical (98.9%) and essential (96.3%) agreement with BMD, with no very major errors. This study is the first VITEK MS–based method that enables rapid detection of colistin resistance in negative ion mode. Colistin resistance was not associated with lipid A modification or mcr‐1–5 genes, suggesting alternative resistance mechanisms, such as efflux pumps.

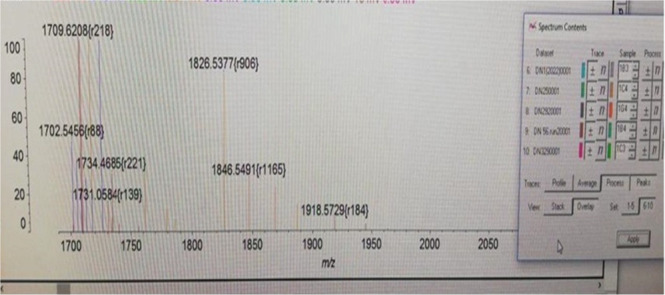

**Abbreviations**
AMR: Antimicrobial Resistance
BMD: Broth Microdilution
BAL: Bronchoalveolar Lavage
CA: Categorical Agreement
CLSI: Clinical and Laboratory Standards Institute
CRAB: Carbapenem‐Resistant *Acinetobacter baumannii*
CSF: Cerebrospinal Fluid
DTA: Distal Tracheal Aspirate
EA: Essential Agreement
EUCAST: European Committee on Antimicrobial Susceptibility Testing
ICU: Intensive Care Unit
ISO: International Organization for Standardization
LPS: Lipopolysaccharide
MALDI‐TOF MS: Matrix‐Assisted Laser Desorption/Ionization Time‐of‐Flight Mass Spectrometry
MDR: Multidrug Resistance
MIC: Minimum Inhibitory Concentration
MMS: MICRONAUT MIC‐Strip
PCR: Polymerase Chain Reaction
pETN: Phosphoethanolamine
VITEK®MS: A MALDI‐TOF Mass Spectrometry System by bioMérieux
VME: Very Major Error

## 1 Introduction

Antimicrobial resistance (AMR) represents a growing global health problem (WHO 2024). In 2019, AMR is a major problem in low‐ and middle‐income countries and is associated with approximately 5 million deaths in 2019. According to the World Health Organization (WHO) 2024 report, carbapenem‐resistant *Acinetobacter baumannii* (CRAB) is identified as the most critical priority pathogen requiring an urgent action plan and the development of novel antibiotics, owing to the paucity of available drug options for treatment (WHO 2024).

Colistin (Polymyxin E) is one of the last‐line antibiotics for the treatment of CRAB, is bactericidal, and is used as monotherapy or in combination with drugs, such as tigecycline, rifampin, and ampicillin/sulbactam (Kumar et al. 2021; Ibrahim et al. 2021). As the use of colistin has increased, colistin susceptibility tests have gained importance and there are difficulties in determining reliable results (Antony et al. 2024).

The phenomenon of colistin resistance in *A. baumannii* strains may be attributable to many distinct mechanisms. First, there may be a complete loss of lipopolysaccharide (LPS) through mutations in LPS‐producing genes (lpxA, lpxB, lpxC, and lpxD). Second, there may be a modification of lipid A components of LPS resulting from mutations in pmrA and pmrB (Lin et al. 2024). Plasmid‐mediated mobile colistin resistance, facilitated by mcr genes, has been documented in numerous *Enterobacterales*, though data concerning *Acinetobacter* species remains limited (Khuntayaporn et al. 2022).

In 2016, the ISO‐20776 standard broth microdilution (BMD) method was recommended for colistin minimum inhibitory concentration (MIC) testing by Clinical and Laboratory Standards Institute and European Committee on Antimicrobial Susceptibility Testing (EUCAST 2016). However, BMD is a time‐consuming and labor‐intensive method for routine clinical microbiology laboratories (Galani et al. 2018). Therefore, an alternative method with satisfactory performance is needed for routine testing. The rapid and reliable determination of colistin susceptibility is important. Recently, several commercially available products based on BMD, which are easy to apply in practice, have been introduced.

On the other hand, matrix‐assisted laser desorption/ionization time‐of‐flight mass spectrometry (MALDI‐TOF MS), which was introduced into clinical microbiology laboratories for rapid identification in the early 2000s, has also been involved in studies aimed at detecting resistance (Dortet, Potron, et al. 2018; Jeannot et al. 2021). Although studies with both Bruker and VITEK MS exist for detecting resistance to various antibiotics, those focused on colistin resistance are primarily conducted with Bruker.

Therefore, in this study, we aimed to compare the results of the commercial kit MICRONAUT‐MIC‐Strip (MMS) with BMD and attempt to analyze the protein peaks of the isolates in negative ion mode using VITEK MS MALDI‐TOF MS. Additionally, we aimed to investigate the presence of *mcr*‐1–5 genotypes in resistant isolates.

## 2 Methods

### 2.1 Bacterial Strain Selection

MDR *A. baumannii* isolates from various samples—including blood, vascular catheter, pleura, cerebrospinal fluid (CSF), wound tissue, abscess, peritoneal fluid, and urine—a total of 194 isolates, sent to the Marmara University Pendik Training and Research Hospital Microbiology Laboratory between December 2020 and 2022, were included in the study. Identification was performed using the VITEK MS (bioMérieux, France) system, and antibiograms were carried out using the disk diffusion (Kirby‐Bauer) method. The first sample of the recurrent strains isolated from the same body part of the same patient was included in the study. The isolates included in the study were stored in skim milk medium at −80°C. Experiments were performed with 194 isolates whose identifications were confirmed.

### 2.2 Carbapenem‐Resistant *A. baumannii*

Isolates with a meropenem zone diameter of less than 15 mm (EUCAST 2016) in routine disc diffusion susceptibility tests and positive for carbapenemase activity via the carbapenemase inactivation method were classified as “carbapenem resistant” and included in the study.

### 2.3 BMD Method

Stock solutions were prepared by suspending the active colistin sulfate (Sigma Aldrich Cat. No. C4461, St. Louis, MO, USA) in accordance with the manufacturer's recommendations. The test was performed in cation‐adjusted Mueller Hinton broth (CAMHB) medium. Serial dilutions between 0.125 and 64 μg/mL were prepared from the stock solution and transferred to microdilution plates. After the suspension of all isolates with a turbidity of 0.5 McFarland standard was prepared, it was added to the microdilution plates as the final bacterial concentration, 5 × 10^5^ CFU/mL. Microplates were incubated at 35°C for 16–20 h. The lowest concentration at which there was no growth was determined as the MIC value for colistin. Results were evaluated by the EUCAST BMD reading guide, and susceptibilities were interpreted using EUCAST breakpoints (EUCAST 2024). *Escherichia coli* ATCC 25922 or *Pseudomonas aeruginosa* ATCC 27853 and *E. coli* NTCC 13846 were used as quality control strains in each microplate.

Colistin susceptibility of *A. baumannii* was classified as susceptible with MIC values ≤ 2 mg/L and resistant with MIC values > 2 mg/L (EUCAST 2016).

### 2.4 Determination of Colistin Susceptibility With MMS

The MMS assay (MERLIN Diagnostika GmbH, Bornheim, Germany) is a single‐isolate strip, containing freeze‐dried colistin in 11 two‐fold dilutions. Each well contains a different colistin concentration ranging from 0.0625 to 64 mg/L. The assay was performed according to the manufacturer's instructions. Bacterial suspensions were prepared to 0.5 McFarland turbidity in saline. Of these, 50 μL was added to the 11 mL of CAMHB. After the mixture was homogenized, 100 μL of the suspension was distributed into the wells and evaluated after 16–20 h of incubation at 35°C. Growth was considered when turbidity was present at the bottom of the well. The tests were considered valid only if growth was observed in the growth control and if no “skipped well” occurred (i.e., no growth in a well but growth in a well with a higher colistin concentration). The first well showing no bacterial growth was defined as the MIC. Essential agreement (EA), defined as the percentage of MICs within ±1 dilution, and categorical agreement (CA), defined as the percentage of test results with the same susceptibility category, were calculated according to ISO standard 20776‐1, using BMD as the reference method for colistin MICs. Results were interpreted based on EUCAST v14 breakpoints (susceptible ≤ 2 mg/L, resistant > 2 mg/L) (ISO 2019). Method agreement was evaluated following ISO 20776‐1 criteria, with EA and CA thresholds of ≥ 90%, and a very major error (VME) rate ≤ 3% (ISO 2007). In addition, Cohen's kappa coefficient was calculated to assess interrater reliability.

### 2.5 MALDIxin Method

A single colony was taken from the isolates, and a suspension was prepared in 2 mL sterile Eppendorf with 200 μL distilled water for injection. For acid hydrolysis, 100 μL of 2% acetic acid was added. The suspension was kept in a heat block at 98°C for 30 min. The suspension was centrifuged at 14.800 rpm for 5 min. The supernatant was removed. Washing with distilled water was performed two more times. After the supernatant was removed, the pellet was mixed with 100 μL distilled water to 20 McFarland turbidity. A 1‐μL of the homogeneous mixture was taken and added on the slide. Pipetting was performed on the slide with 2 μL of 2,5‐dihydroxybenzoic acid matrix (Sigma Aldrich Cat. No. 85707, Gillingham, UK). The mixture was spread on the slide, dried in air flow for 2 min and loaded into the system.

After calibration settings, MALDI‐TOF MS analysis was performed using RUO saramis 4.19 (VITEK MS, bioMérieux, France) in negative ion mode with raster type regular circular, well shape circular, diameter 1600, spacing 150. Laser ignition was set to power 80, profiles 100, shots 10, laser rep rate 50, ion gate 300 in linear saramis mode, and 200–1000 laser shots were performed, and data were collected in 500–4000 spectra. The analyses were performed using an MS database with mzxml and txt file records.

### 2.6 Detection of *mcr* Genes

Detection of mcr‐1–5 genes was performed as described by Lescat et al. (2018). Briefly, *A. baumannii* strains were analyzed for the presence of mcr‐1–5 genes using multiplex polymerase chain reaction (PCR). For DNA isolation, colonies of bacteria grown overnight were used. The colonies were put in a test tube containing 500 µL of distilled water and boiled for 10 min in a water bath, and then centrifuged at 10,000*g* for 5 min. PCR for each isolate was performed in a total volume of 25 μL, using a master mix consisting of 12.5 μL buffer solution (GM SYBR qPCR Kit, Genemark Cat. No. QPSY02‐1, Türkiye), 5 μL DNA polymerase (Promega, USA), 0.2 mM deoxyribonucleotide triphosphate mix (Ampliqon, Denmark), 0.5 μM primer (Oligomer, Türkiye), 1.5 mM MgCl_2_, and 2 μL template DNA. The primers used for PCR are listed in Table 1.

**Table 1:** Primers used for genotypic analysis.

Primers	Sequence (5′–3′)	Amplicon size (bp)
CLR F CLR R Mcr‐2 F Mcr‐2 R Mcr‐3 F Mcr‐3 R Mcr‐4 F Mcr‐4 R Mcr‐5 F Mcr‐5 R	F‐CGG TCA GTC CGT TTG TTC R‐CTT GGT CGG TCT GTA GGG F‐TGT TGC TTG TGC CGA TTG GA R‐AGA TGG TAT TGT TGG TTG CTG F‐AAA TAA AAA TTG TTC CGC TTA TG R‐AAT GGA GAT CCC CGT TTT T F‐TCA CTT TCA TCA CTG CGT TG R‐TTG GTC CAT GAC TAC CAA TG F‐ATGCGGTTGTCTGCATTTATC R‐TCATTGTGGTTGTCCTTTTCTG	309 567 929 1116 1644

The amplification process (T100 Thermal Cycler, Bio‐Rad) involved an initial denaturation at 94°C for 15 min, followed by denaturation at 94°C for 30 s, annealing at 58°C for 90 s, polymerization at 72°C for 60 s (25 cycles), and final elongation at 72°C for 10 min. The products were loaded onto a 1.5% agarose gel containing 0.5 μg/mL ethidium bromide and observed in a ultraviolet (UV) transilluminator following electrophoresis at 110 V.

## 3 Results

A total of 194 *A. baumannii* isolates from clinical specimens received between December 2020 and 2022 were included in the study. Of the samples, 56.1% were respiratory samples (bronchoalveolar lavage [*n*:4] 2.06%, deep tracheal aspirate‐endotracheal aspirate [*n*:88] 45.3%, sputum [*n*:15] 7.7%, deep pharyngeal swab [*n*:2] 1.03%), while other sample types included blood (*n*:55) 28.3%, vascular catheter (*n*:1) 0.5%, pleura (*n*:3) 1.5%, CSF (*n*:3) 1.5%, wound tissue (*n*:8) 4.1%, abscess (*n*:3) 1.5%, peritoneal fluid (*n*:4) 2.06%, and urine (*n*:8) 4.1%. Of these samples, 97.4% (*n*:189) were from inpatients and 2.6% (*n*:5) from outpatients.

**Figure 1:**
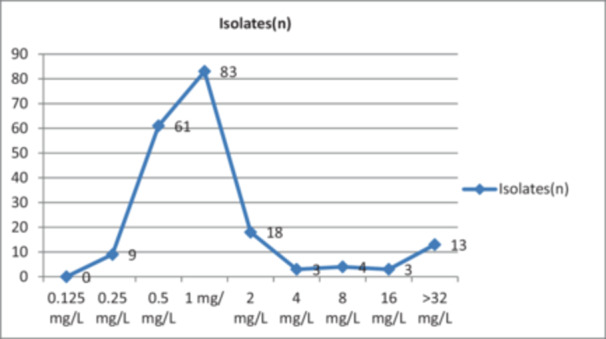
Minimum inhibitory concentration distribution of *Acinetobacter baumannii* strains.

Of 194 *A. baumannii* isolates analyzed by BMD, 23 (11.8%) were resistant to colistin. The MIC_50_ value of the isolates was 1 mg/L, while the MIC_90_ value was 2 mg/L. Of the resistant isolates, 39.1% was isolated from respiratory specimens and 17.3% was found to be causative agents of bloodstream infections. Colistin MIC was > 16 mg/L in 11% of respiratory samples. Among the colistin‐susceptible isolates, 42.7% had MIC, 1 mg/L; 31.4% had MIC, 0.5 mg/L; 9.2% had MIC, 2 mg/L (Figure 1).

In Figure 1, it is observed that the majority of susceptible isolates had MIC values < 2 mg/L, while most of the resistant isolates had MIC values ≥ 8 mg/L. Considering the susceptibility threshold of 2 mg/L, most of the resistant isolates were found to have high MIC values.

Compared with the reference method, CA was 98.9% and EA was 96.3% in MMS. When the MIC distributions were evaluated, no VME was detected and the ME rate was determined as 1.03%. The study found an excellent agreement between the MMS and BMD methods, with Cohen's kappa coefficient value of 0.97.

The MIC distribution of all isolates determined by the BMD and MMS test is given in Table 2.

**Table 2:** MIC distributions of all isolates determined by broth microdilution (BMD) method and MICRONAUT‐MIC‐Strip test.

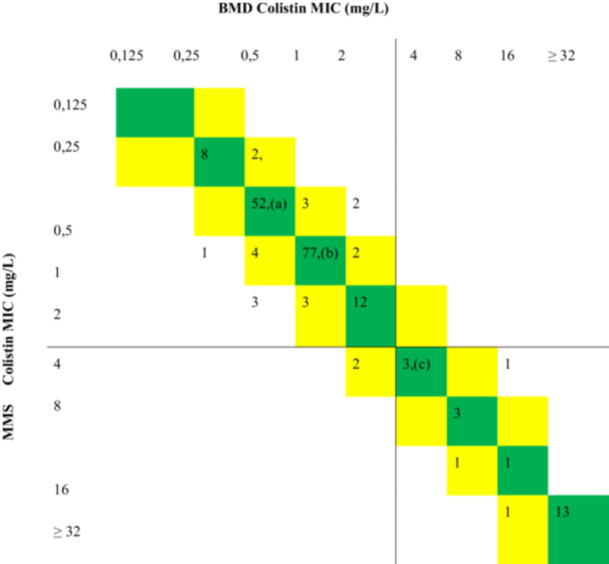

*Note:* Green squares signify identical values, and yellow squares signify values within ±1 dilution of BMD MICs. The black lines indicate the EUCAST clinical breakpoints (susceptible ≤ 2 mg/L, resistant > 2 mg/L). The quality control strains included are: (a) *Escherichia coli* ATCC 25922 (MIC 0.5 mg/L), (b) *Pseudomonas aeruginosa* ATCC 27853 (MIC 1 mg/L), and (c) *E. coli* NCTC 13846 (MIC 4 mg/L). All quality control strains were tested in duplicates, resulting in the same MIC value both times.

Abbreviations: EUCAST, European Committee on Antimicrobial Susceptibility Testing; MIC, minimum inhibitory concentration; MMS, MICRONAUT‐MIC‐Strip.

**Figure 2:**
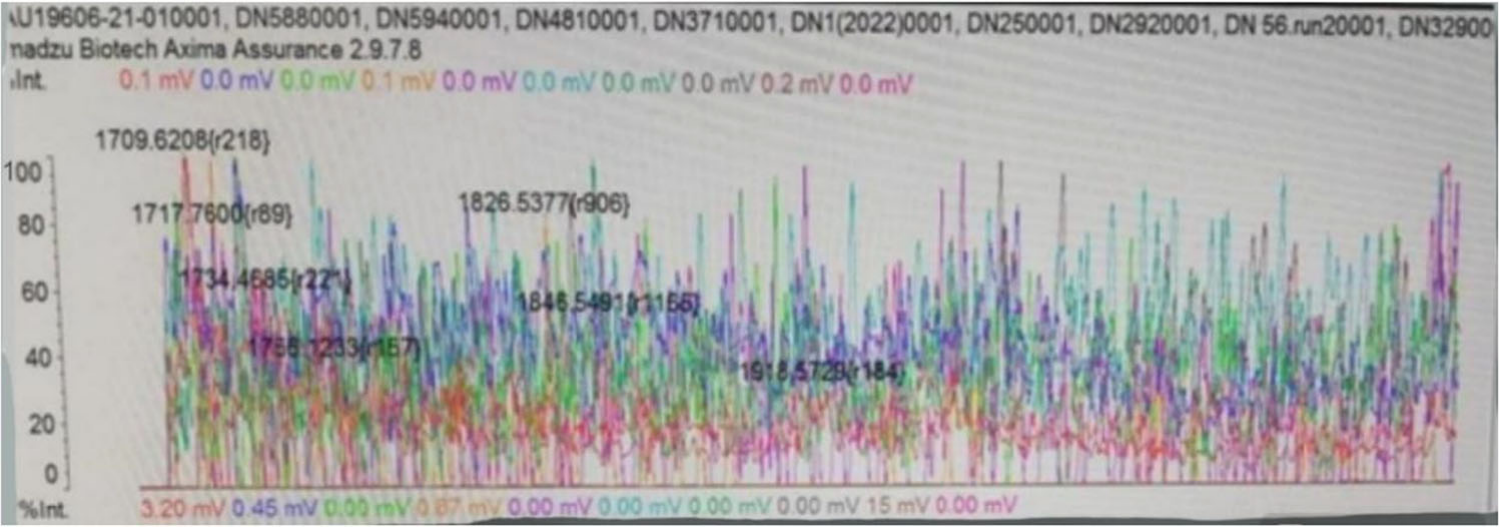
Distribution of lipid A peaks (1690–2137 *m*/*z*) in *Acinetobacter baumannii* Isolates: Assessment of colistin resistance via the MALDIxin method.

**Figure 3:**
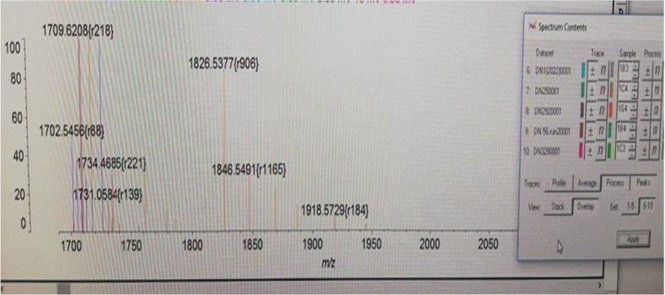
Matrix‐assisted laser desorption/ionization time‐of‐flight mass spectrometry spectrum of lipid A extracted from colistin‐susceptible *Acinetobacter baumannii* ATCC 19606, showing the expected dominant peak at 1918 *m*/*z*.

**Figure 4:**
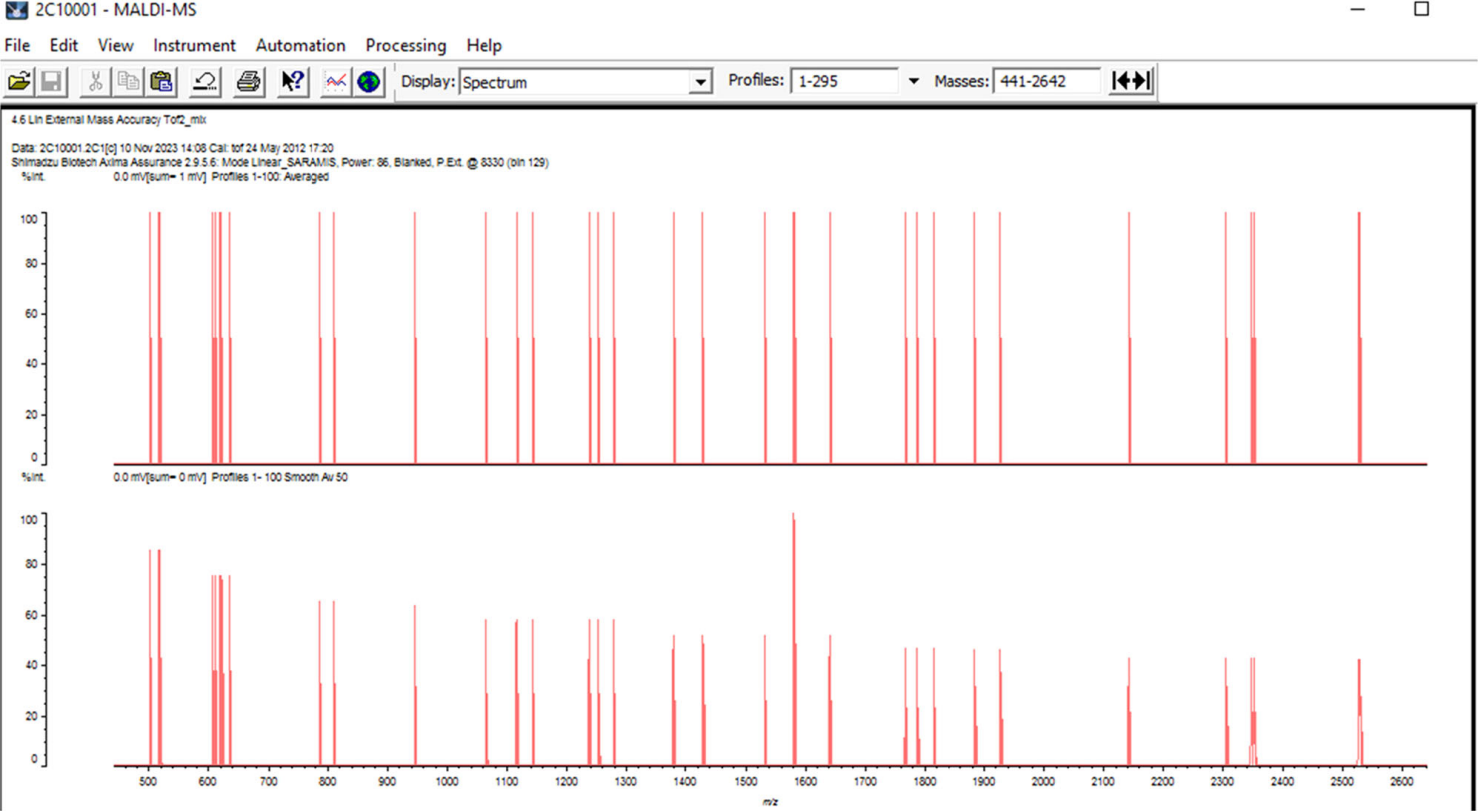
The figure illustrates the MALDIxin method of *Escherichia coli* LPS O55:B5 obtained, with a characteristic peak observed at 1796 *m*/*z*. LPS, lipopolysaccharide.

By the MALDIxin method, lipid A peaks in colistin‐susceptible and resistant isolates remained between 1730 and 1910 *m*/*z*, while the peak at 1728 *m*/*z* of colistin‐susceptible *Acinetobacter baumannii* 19606 was observed, where it was expected to be (Figures 2 and 3). Images of the peaks formed at 1796 *m*/*z* by the MALDIxin method of *E. coli* LPS O55B5 are given in Figure 4.

**Figure 5:**
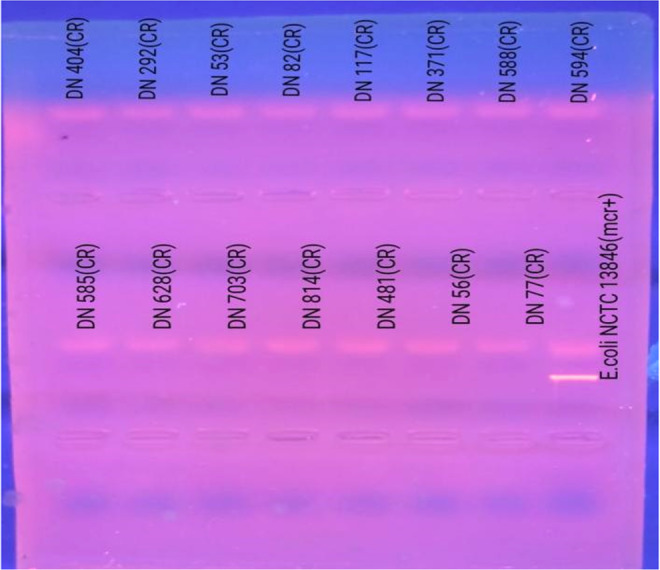
The results of mcr‐1, mcr‐2, mcr‐3, mcr‐4, and mcr‐5 PCR in colistin‐resistant clinical isolates and *Escherichia coli* NCTC 13846 on gel electrophoresis. PCR, polymerase chain reaction.

The mcr‐1–5 genes responsible for colistin resistance were analyzed by PCR, but no gene was found in any of the isolates. The UV transluminator images of the amplification products of some of these isolates in gel electrophoresis are given in Figure 5.

## 4 Discussion

*Acinetobacter calcoaceticus–baumannii* complex infections are typically seen in ventilator‐dependent, catheter‐associated patients with open wounds, especially in patients hospitalized in intensive care units (ICUs) with prolonged hospitalization (Ibrahim et al. 2021). In a study with 136 *A. baumannii* and six *Acinetobacter lwoffii* isolates, it was reported that 64% of the samples sent to the laboratory were isolated from patients hospitalized in the ICU. It was reported that half of the patients were associated with mechanical ventilation or central venous catheter (Liu et al. 2022; Elham and Fawzia 2019). Consistent with the literature, 78.8% (*n* = 153) of the isolates in our study were isolated from the ICU.

The prevalence of CRAB varies geographically, with studies reporting an increase in colistin resistance in CRAB isolates over the last two decades (Bostanghadiri et al. 2024). A meta‐analysis reported that there were differences in colistin resistance in different geographical regions, such as with colistin resistance rates of 7% in Western Europe, 19% in Iraq, and 18% in Greece (Bostanghadiri et al. 2024).

In our study, colistin resistance of CRAB isolates was found to be 11.8% with the reference method BMD. In the literature, there are many studies investigating CRAB strains. The meta‐analysis encompassed a range of studies that investigated the prevalence of colistin resistance. Similar to our results, Jia et al. (2020) found colistin resistance in *A. baumannii* isolates to be 13.7% (8/58) by the BMD test. In another study investigating colistin susceptibility of CRAB isolates, colistin resistance was reported as 52.9% (9/17) (Fam et al. 2020).

The fact that the BMD test is troublesome for routine clinical microbiology laboratories leads to the search for alternative commercial methods for this purpose. For this purpose, many commercial kits are produced and studies are carried out.

Matuschek et al. (2018) employed the BMD method as a reference and investigated the performance of the MMS method and other commercial kits. In the study conducted with a total of 75 MDR isolates, 100% CA and ≥ 96% EA with the MMS method were found, suggesting that this method can be used for colistin susceptibility. However, a survey of the literature on the performance of commercial kits yielded divergent results. Yusuf et al. conducted a comparative analysis of four commercially available kits designed to assess colistin susceptibility, utilizing the BMD reference method as a standard. Their findings revealed that the CA and EA of the MMS method were 96% and 83%, respectively (Yusuf et al. 2020). Kon et al. investigated the performance of MMS with a total of 273 strains, including CRAB and carbapenem‐resistant *Enterobacterales* isolates. They found that MMS had 98.5% sensitivity and 99.5% specificity. They suggested that the commercial MMS kit can be used as an alternative to BMD for colistin susceptibility testing (Kon et al. 2021).

In our study, the CA of the MMS test was 98.9% and the EA was 96.3%. Cohen's kappa coefficient for the MMS method was determined as 0.97, indicating excellent agreement. MMS test showed MIC 4 mg/L (resistant) in two strains with MIC 2 mg/L (susceptible) by the reference method. Considering the acceptable ±1 dilution range. We think that the MMS method is applicable for the determination of colistin susceptibility. Studies with MMS have indicated that measurement variability is more likely near the susceptibility breakpoint, similar to our findings (Matuschek et al. 2018; Kon et al. 2021). In addition, an important advantage of this method is that there are removable wells for each patient, so that susceptibility can be determined separately for each isolate.

Recent advancements in proteomics have led to a significant increase in the rapid detection of antibiotic resistance. In addition to the extensive utilization of MALDI‐TOF MS for the expeditious identification of microorganisms, recent studies have been conducted to detect antibiotic resistance profiles based on protein peaks (Horseman et al. 2020).

The MALDI‐TOF‐based method for the detection of colistin resistance, termed MALDIxin, was first developed by Dortet et al. The mass spectrum of sensitive *A. baumannii* strains is characterized by two distinct peaks at 1728.1 and 1910.3 *m*/*z*. In colistin‐resistant (CR) strains, the mass spectrum is dominated by two sets of peaks centered at 1935.3 and 2033.3 *m*/*z*, corresponding to the +25 and +123 *m*/*z* shifts previously observed in the mass unit of native bis‐phosphorylated hepta‐acyl lipid A at 1910.3 *m*/*z* (Supp file). The addition of a phosphoethanolamine (pETN) moiety to the phosphate group at position 1 leads to a shift of +123 *m*/*z* in the peak corresponding to native lipid A, while the addition of a pETN moiety to 4′ of native lipid A leads to a shift of +25 *m*/*z* in the peak corresponding to native lipid A, accompanied by the loss of the phosphate group at position 1 (Dortet, Potron, et al. 2018). It is possible to determine the mechanisms responsible for colistin resistance in a very short time with rapid methods using MALDI‐TOF mass spectrometry of the change in lipid A structure (Furniss et al. 2020; Tang et al. 2023).

Dortet et al. applied the MALDIxin method to 46 isolates and found shifts in protein peaks in three isolates. In another study, protein peaks at 1935 and 2033 *m*/*z* were detected by the MALDIxin method in nine CR and eight colistin‐susceptible *A. baumannii* isolates (Dortet, Potron, et al. 2018; Dortet, Bonnin, et al. 2018).

We aimed to detect colistin resistance with the MALDIxin procedure and the method developed in VITEK MS RUO saramis negative ion mode. Negative ion mode is not used in routine applications. This method was applied to isolates thawed from −80°C stock. The expected peaks in resistant isolates were 1935 and 2033 *m*/*z*. However, none of the isolates showed these peaks. We interpret this result as lipid A modification was not responsible for the colistin resistance of our isolates. Other potential explanations may include the loss of resistance in isolates following storage and revival from stock, or the influence of the limitations associated with the negative ion mode detection in the VITEK MS system.

Larrouy‐Maumus et al. 90 *E. coli* isolates investigated the mechanisms responsible for colistin resistance with a commercial MALDI‐TOF MS–based kit. They suggested that false positivity and false negativity may occur with the MALDIxin method because of inducible mcr‐1 gene mutations and/or plasmid‐induced resistance may be lost in strains stored and thawed at −80°C, and therefore, isolates isolated from clinical samples should be studied as soon as possible (Larrouy‐Maumus et al. 2023). In our study, the inability to detect resistance mechanisms using the MALDIxin method may be attributed to the use of previously frozen and subsequently recultivated isolates. Additionally, the limited resolution of the VITEK MS system in negative ion mode may have also contributed to this outcome.

Recent studies have pointed to the potential for determining colistin resistance status in the positive ion mode, a development with important implications for routine laboratory practice. They suggested that the so‐called “CORE” method (which qualitatively and quantitatively determines colistin susceptibility in positive ion mode and can provide results within 3 h) is cost‐effective and may be effective in preventing the spread of antibiotic resistance. Another method for rapid antibiotic susceptibility results and detection of resistance genes based on MALDI‐TOF MS is DOT‐MGA (direct‐on‐target microdroplet growth assay). The authors posited that further research is required to standardize and optimize these methodologies (Foglietta et al. 2023; Li et al. 2021; Neonakis and Spandidos 2019). To the best of our knowledge, the studies in the literature with MALDI‐TOF MS in negative ion mode were performed with Bruker (Bruker Daltonics, Germany) and our study is the first study with VITEK MS (bioMérieux, France) in negative ion mode. Further studies are needed in this field. We believe that this method we developed will shed light on future studies.

The plasmid‐mediated mcr gene was first identified in China and has rapidly spread among Gram‐negative bacteria, with reports from over 60 countries to date. In the literature, a study conducted on 82 *A. baumannii* isolates investigated the presence of mcr genes, and it was reported that none of the isolates carried these genes (Germ et al. 2019). In our study, the presence of mcr‐1–5 genes, which cause acquired colistin resistance, was also investigated. However, these genes could not be detected. We attribute this to the rarity of colistin resistance mediated by mcr genes in non‐fermenting bacteria or to the involvement of other resistance mechanisms in our isolates. Previous studies conducted in our country have reported that colistin resistance attributed to mcr genes is not widespread.

Our study has some limitations. First, the number of CR isolates is relatively limited. Second, mcr‐mediated colistin resistance was not detected. Third, whole genome sequencing was not performed. Since neither mcr‐1–5 genes nor lipid A modifications were detected, WGS could have revealed chromosomal mutations or efflux pump involvement. Finally, the MALDI‐TOF method was performed on isolates that were stored and revived.

## 5 Conclusion

Rapid detection of colistin resistance for CRAB isolates posing an immediate threat is of great importance for routine clinical microbiology laboratories. In this sense, the results of our study show that the MMS test can be used with a CA of 98.9% and an EA of 96.3%.

Effective detection of resistance and the spread of microorganisms to antibiotics is crucial for preventing nosocomial infections. Given that colistin is the last‐resort antibiotic, and the indiscriminate use of antibiotics contributes to the rise of resistant strains, it is essential to use colistin with great caution.

## Author Contributions

**Fatih Mehmet Akıllı:** conceptualization, investigation, writing – original draft, writing – review and editing, visualization, validation, methodology, software, formal analysis, resources, supervision, data curation, project administration. **Arzu İlki:** conceptualization, investigation, funding acquisition, writing – original draft, writing – review and editing, visualization, validation, methodology, software, formal analysis, project administration, resources, supervision, data curation.

## Ethics Statement

Ethical approval for the manipulation of bacterial strains in this study was obtained from Marmara University Ethics Committee (Approval Code 09.2022.1051).

## Conflicts of Interest

The authors declare no conflicts of interest.

## Supporting information

Supplement 1.

## Data Availability

The authors have nothing to report.
