# Supplementary figures and images for: Evaluation of Colistin Susceptibility in Carbapenem‐Resistant Acinetobacter baumannii Isolates Using Broth Microdilution, MICRONAUT‐MIC‐Strip, and VITEK MS
Source: Microbiologyopen. 2025 Aug 8;14(4):e70046. doi: 10.1002/mbo3.70046 (PMC12332535; doi:10.1002/mbo3.70046)

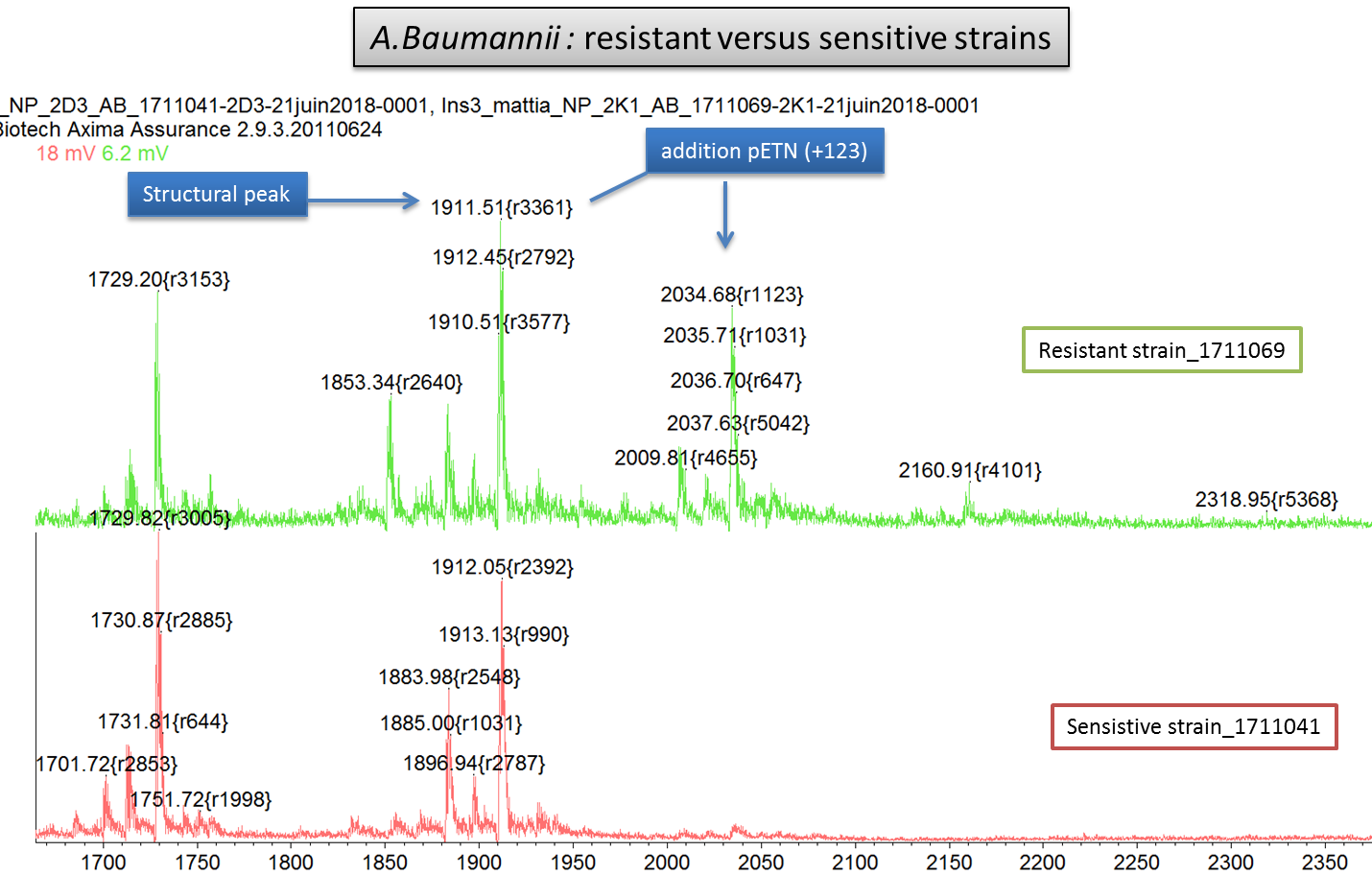

Supplement: Supplementary file 1 — Supplement 1. [file MBO3-14-e70046-s001.docx]
